# Risk of gout attack not increased in patients with thalassemia: a population-based cohort study

**DOI:** 10.1038/s41598-023-29709-3

**Published:** 2023-02-16

**Authors:** Jing-Wen Chen, Qiang Xu, Pei-Dan Yang, Jing-Yang Huang, James Cheng-Chung Wei

**Affiliations:** 1 Department of Rheumatology, the First Affiliated Hospital of Guangzhou University of Chinese Medicine, Guangzhou, 510405 China; 2 Guangzhou University of Chinese Medicine, Guangzhou, 510405 China; 3 Department of Medical Research, Chung Shan Medical University Hospital, Taichung, 40201 Taiwan; 4 Department of Allergy, Chung Shan Medical University Hospital, Immunology & Rheumatology, Taichung, 40201 Taiwan; 5 Institute of Medicine, College of Medicine, Chung Shan Medical University, Taichung, Taiwan; 6 Graduate Institute of Integrated Medicine, China Medical University, Taichung, Taiwan

**Keywords:** Rheumatology, Musculoskeletal system

## Abstract

The incidence of gout arthritis in patients with thalassemia and the association between them was indefinite. We aimed to give epidemiological evidence regarding the association between thalassemia and gout arthritis. This retrospective cohort study extracted data relating to the risk of gout arthritis from patients diagnosed with thalassemia between 2000 and 2013. We selected the control group at a ratio of 1:4 by propensity score matching (PSM). Univariable and multivariable Cox proportional hazard regression models were performed to analyze the association between thalassemia and gout arthritis and to evaluate the hazard ratio (HR) of gout arthritis after exposure with thalassemia. The sensitivity analysis was performed to avoid the mislabeled thalassemia disease, the transfusion-dependent thalassemia was classified to compare the risk of gout arthritis. The secondary outcome for the risk of gout arthritis with antigout drugs treatment was also evaluated between study groups. In the age and sex matched cohort, the majority of thalassemia patients were women (62.03%) and aged younger than 30 years old (44.79%). There were 138 (4.2%) and 500 (3.8%) incident cases of gout arthritis in the thalassemia and non-thalassemia group. After PSM, the incidence rate, per 100 person-years, of gout arthritis was 0.48 (95% CI 0.42 to 0.56) and 0.60 (95% CI 0.51 to 0.72) in non-thalassemia individuals and patients with thalassemia, respectively. In the Cox proportional hazard regression, patients with thalassemia had no significant increase in the risk of gout arthritis (adjusted HR, 1.00; 95%CI: 0.80 to 1.25) after adjusting demographic variables and comorbidities. The Kaplan–Meier curve showed that the cumulative incidence of gout arthritis was not a significant difference in the thalassemia group than in the comparison group (p > 0.05). The sensitivity analysis showed the consistent results about the risk of gout arthritis in patients with thalassemia. Our study indicated that there was no significant increase in the risk of gout arthritis in subjects with thalassemia.Future research needs to clarify the biological mechanisms behind this connection.

## Introduction

Thalassemia was a hemoglobin (Hb) disturbance characterized by a deficiency in the synthesis of one or more globulin subunits of hemoglobin^1^. This defection resulted in an imbalance in the α/β globin chain, ineffective erythropoiesis, chronic hemolytic anemia, and iron overload. They were classified as the types α, β, δβ, and δβγ-thalassemias. Among them α and β-thalassemias were the most common types clinically^2^. Currently, based on their clinical severity and transfusion requirement, these thalassemia syndromes were classified phenotypically into two main groups: Transfusion Dependent Thalassaemias and Non-Transfusion Dependent Thalassaemias^3^. Clinically, Diagnosis of thalassemia and hemoglobinopathies required a comprehensive evaluation combining clinical manifestations red blood cell phenotypes, hemoglobin profiles, and DNA analysis: (1) Haematological examination: If mean corpuscular volume (MCV) < 80 fl and/or mean corpuscular hemoglobin (MCH) < 27 pg, participants were viewed as potential carriers of thalassemia. Potential thalassemia carriers were sustained to further genetic examination; (2) Genetic screening: Three genes (HBA1, HBA2, and HBB) were closely related to α-thalassemia and β-thalassemia according to DNA analysis^4^.

Gout was aroused by long-term hyperuricemia which results in the sedimentation of monosodium urate (MSU) crystals that accumulate in joints and other tissues^5^. Distinguished as the most common form of inflammatory arthritis, the effective marker for gout diagnosis was the spotting of MSU crystals in synovial fluid. Rheumatological manifestations ranging from musculoskeletal complications to connective tissue diseases are common among thalassaemia patients.The correlation between thalassemia andsome rheumatological and non-rheumatological autoimmune disorders is worthy of the attention of rheumatologists, as there may be significant differences in prognosis and treatment^6^. To our knowledge, the association between thalassemia and gout arthritis was rarely reported^7^. A few studies indicated that patients with thalassemia probably suffered from crystal arthritis, and suggested that gout and thalassemia were a rare but significant association^8–11^. Thalassemia patients were more likely to develop uric acid deposition disease in case of just slightly abnormal renal function, which resulted from an increased rate of red blood cell destruction and high levels of endogenous as well as blood uric acid^12^. However, these published researches were essentially case-reports or cross-sectional design, which was no control group and sufficient sample size to systematically assess the association between thalassemia and gout. Therefore, we aimed to investigate the incidence of gout arthritis among patients with and without thalassemia and analyze this association by reviewing a databank of 1 million population.

## Patient and methods

### Data sources and study design

We designed a retrospective cohort study using the claims data from the Longitudinal Health Insurance Database 2000 (LHID 2000), Taiwan, which is a social health insurance system enrolls approximately 99% of Taiwan’s population. The patients' demographic characteristics, clinical manifestations, diagnosis, and treatment have been recorded in this database^13^. All methods were performed in accordance with relevant guidelines and regulations.

This study was approved by Chung Shan Medical University Hospital RB​, CS15134. Informed consent was waived by Chung Shan Medical University Hospital IRB, since this is a population-based retrospective cohort study that following Taiwan’s Personal Information Protection Act, and the anonymity of the database.

### Study participants

**Figure 1:**
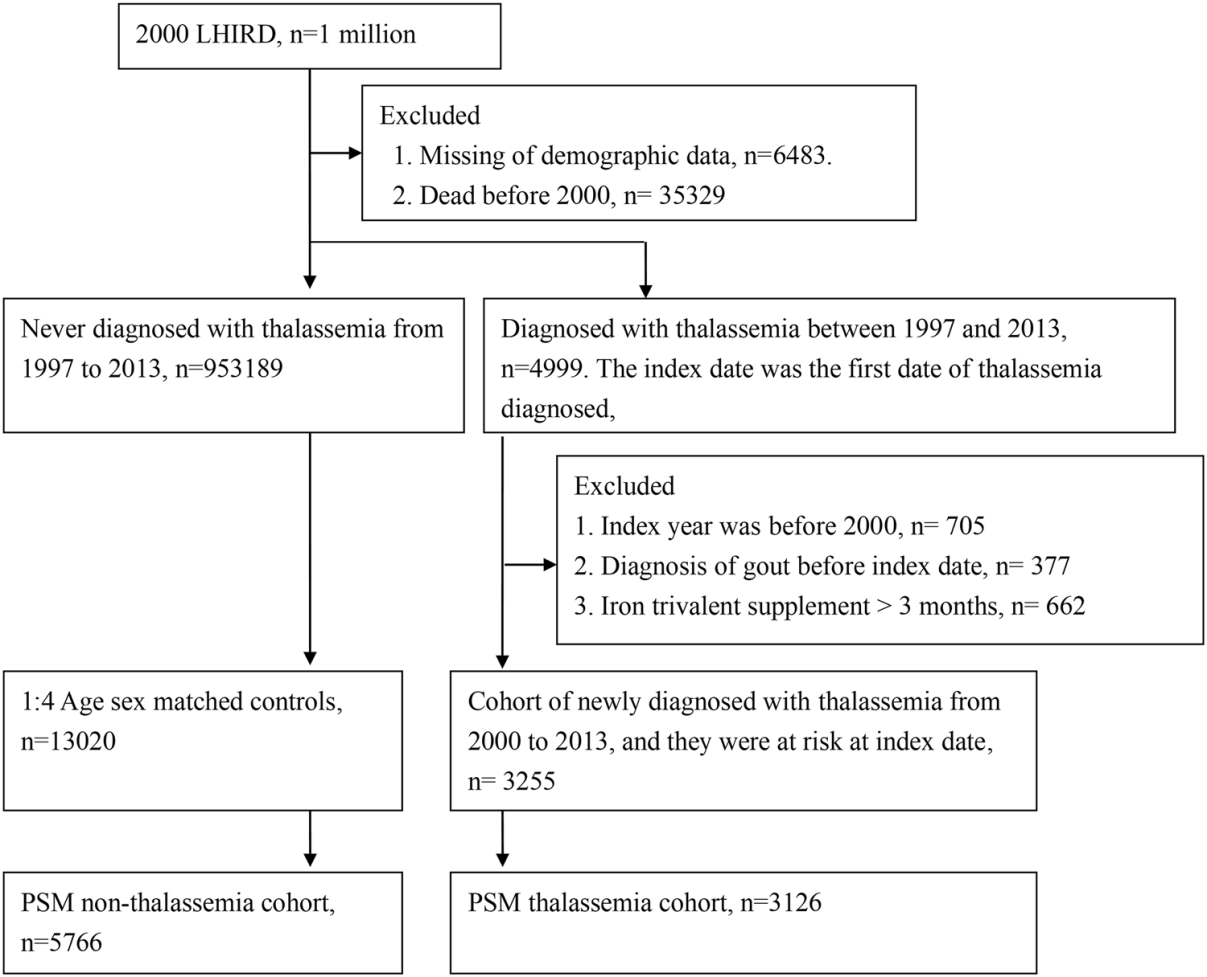
Study flow chart.

LHID 2000 comprised one million people that were sampled from Taiwan national health insurance research datasets^14^. Diseases were classified with the International Classification of Diseases, Ninth Revision Clinical Modification (ICD-9-CM). The patients, who were newly diagnosed with thalassemia (ICD-9-CM code 282.4) from 2000 to 2013, were identified from LHID 2000. In order to improve the accuracy of the diagnosis of thalassemia, the patients who had more than 2 outpatient visits or at least 1 hospitalization for thalassemia were defined as the thalassemia cohort, and the first date of thalassemia diagnosis was defined as the index date. The exclusion criteria included: (1) index year was before 2000 (n = 705); (2) patients were diagnosed with gout before index date (n = 377); (3) the patients had iron trivalent supplement with more than 3 months (if thalassemia patients is taking iron for a long period of time, then the diagnosis of thalassemia may not be valid, n = 662). Finally, a total of 3255 patients was included for primary analysis (Fig. 1).

### Non-thalassemia control group

In the LHID 2000, there were 953,189 subjects had never been diagnosed with thalassemia from 1997 to 2013. Subsequently, we matched thalassemia patients with non-thalassemia individuals at a ratio of 1:4 by gender and age, there were 13,020 non-thalassemia individuals, who had the same index date with matched thalassemia patient and they were all at risk on the index date.To minimize the influence of confounding bias, we used a propensity score matching (PSM) to balance the baseline co-variate between study groups.The propensity score of patients with thalassemia was calculated by logistic regression using PROC PSMATCH under SAS software. Each thalassemia patient was 1:2 matched with individuals without thalassemia by the propensity score calculated using demographics (including age, sex, urbanization, insured unit), length of hospital stay, and co-morbidities (including rheumatoid arthritis, Sjogren’s syndrome, systemic sclerosis, vasculitis, hypertension, diabetes mellitus, hyperlipidemia, coronary artery disease, osteoporosis, stroke, asthma, chronic obstructive pulmonary disease, chronic kidney disease, chronic liver diseases, hyperthyroidism, thyroiditis, pancreatitis, affective psychosis, ankylosing spondylitis, inflammatory bowel disease, human immunodeficiency virus infection, and antiphospholipid antibody syndrome) at baseline by the nested greedy algorithm with the caliper of 0.01. As a result, PSM identified 3126 thalassemia patients and 5766 non-thalassemia patients (Fig. 1).

### Outcome and covariates

Patients diagnosed with gout arthritis (ICD-9-CM code 274.0) with more than 2 outpatient visits or at least one hospitalization were defined as the primary outcome. Demographic data analyzed in this study were age at index date, sex, urbanization, various insured units. The comorbidities included hypertension (ICD-9-CM codes 401–405), diabetes mellitus (ICD-9-CM code 250), hyperlipidemia (ICD-9-CM codes 272.0–272.4), coronary artery disease (ICD-9-CM codes 410–414), osteoporosis (ICD-9-CM code 733), rheumatoid arthritis (ICD-9-CM code 714.0), Sjogren’s syndrome (ICD-9-CM code 710.2), systemic sclerosis (ICD-9-CM code 710.1), vasculitis (ICD-9-CM code 443.0), stroke (ICD-9-CM codes 430–438), asthma (ICD-9-CM code 493), chronic obstructive pulmonary disease (ICD-9-CM codes 490–492 and 493–496), chronic kidney disease (ICD-9-CM code 585), chronic liver diseases (ICD-9-CM codes 571 and 573), hyperthyroidism (ICD-9-CM code 242), thyroiditis (ICD-9-CM code 245)^15^.

### Sensitivity analysis

We performed the sensitivity analysis to discuss the potential bias in this study. To avoid the mislabeled thalassemia disease, the transfusion-dependent thalassemia was defined as the patients had catastrophic illness certificate of thalassemia, or receiving regular transfusion and iron-chelating agents (reference: Pediatr Blood Cancer 2017; 64: 135–138). Furthermore, we defined the secondary outcome as the patient had the diagnosis of gout arthritis (ICD-9-CM code 274.0) and receiving antigout drugs, involving colchicine, cinchophen, allopurinol, febuxostat, and benzbromarone. In these alternative scenarios, we increased the positive predictive value when defining the patients had thalassemia disease and gout arthritis.

### Statistical analysis

We conducted an absolute standardized difference (ASD) to assess the balance of baseline variables in the age and sex matched cohorts and PSM cohorts. In general, ASD < 0.10 indicates that the variable is balanced between the two groups. The Poisson regression model was used in estimating the incidence rate (per 100 patients-year) of gout arthritis and crude relative risk (and its 95%CI). We draw a Kaplan–Meier curve to observe the cumulative incidence of gouty arthritis since the index date. The log-rank test showed that the difference in the cumulative incidence of gouty arthritis between the thalassemia group and the controls when p < 0.05. Two models, the univariate model, and multivariate Cox proportional hazard regression were used to estimate the hazard ratio (HR) and 95% CI of gout arthritis for thalassemia exposure and covariates. Our data were analyzed using SAS 9.4 version software and p < 0.05 (2-sided) was considered statistically significant.

## Results

### Demographic characteristics and comorbidities in thalassemia and non-thalassemia group

The control group was firstly matched by sex and age at a ratio of 1:4. Eligible subjects were 3255 patients in the thalassemia group and 13,020 in the non-thalassemia group. Subsequently, we used PSM to minimize the influence of confounding variables. As a result, 3126 patients with thalassemia and 5766 non-thalassemia cohort after PSM were collected (Fig. 1). In the age-matched and sex-matched cohort, most of the thalassemia participants were female (62.03%) and younger (44.79%). The incidence rate of comorbidities, namely chronic liver diseases (12.53%) , hypertension (12.29%), affective psychosis (10.84%), COPD (7.90%), diabetes mellitus (7.59%), and hyperlipidemia (7.59%) was higher in the thalassemia cohort. The PSM analysis revealed that baseline characteristics were generally balanced (ASD < 0.1) (Table 1).

**Table 1:** Characteristics among thalassemia groups and non-thalassemia groups.

	Before PSM			After PSM		
	Non-thalassemia n = 13,020	Thalassemia n = 3255	ASD	Non-thalassemia n = 5766	Thalassemia n = 3126	ASD
Age at index date			0.00			0.03
< 30	5842 (44.87%)	1458 (44.79%)		2646 (45.89%)	1408 (45.04%)	
30–45	3483 (26.75%)	876 (26.91%)		1536 (26.64%)	845 (27.03%)	
45–60	2141 (16.44%)	536 (16.47%)		894 (15.50%)	512 (16.38%)	
≥ 60	1554 (11.94%)	385 (11.83%)		690 (11.97%)	361 (11.55%)	
Sex			0.00			0.02
Female	8076 (62.03%)	2019 (62.03%)		3708 (64.31%)	1961 (62.73%)	
Male	4944 (37.97%)	1236 (37.97%)		2058 (35.69%)	1165 (37.27%)	
Urbanization			0.05			0.00
Urban	8042 (61.77%)	1937 (59.51%)		3420 (59.31%)	1857 (59.40%)	
Sub-urban	3781 (29.04%)	1018 (31.27%)		1787 (30.99%)	979 (31.32%)	
Rural	1197 (9.19%)	300 (9.22%)		559 (9.69%)	290 (9.28%)	
Insured unit			0.05			0.08
Public insurance	926 (7.11%)	264 (8.11%)		516 (8.95%)	253 (8.09%)	
Labour insurance	8121 (62.37%)	2025 (62.21%)		3511 (60.89%)	1947 (62.28%)	
Agricultural insurance	1690 (12.98%)	429 (13.18%)		782 (13.56%)	412 (13.18%)	
Low-income household	69 (0.53%)	23 (0.71%)		47 (0.82%)	22 (0.70%)	
Company insurance	1866 (14.33%)	439 (13.49%)		758 (13.15%)	419 (13.40%)	
Other	348 (2.67%)	75 (2.30%)		152 (2.64%)	73 (2.34%)	
Length of hospital stay			0.32			0.09
0	12,080 (92.78%)	2694 (82.76%)		4979 (86.35%)	2626 (84.01%)	
1–6	629 (4.83%)	310 (9.52%)		521 (9.04%)	290 (9.28%)	
≥ 7	311 (2.39%)	251 (7.71%)		266 (4.61%)	210 (6.72%)	
Co-morbidities						
Rheumatoid arthritis	65 (0.50%)	40 (1.23%)	0.08	58 (1.01%)	35 (1.12%)	0.01
Sjogren’s syndrome	44 (0.34%)	18 (0.55%)	0.03	29 (0.50%)	16 (0.51%)	0.00
Systemic sclerosis	0 (0.00%)	1 (0.03%)	0.03	0 (0.00%)	0 (0.00%)	0.00
Vasculitis	10 (0.08%)	9 (0.28%)	0.05	9 (0.16%)	8 (0.26%)	0.02
Hypertension	1090 (8.37%)	400 (12.29%)	0.13	701 (12.16%)	370 (11.84%)	0.01
Diabetes mellitus	511 (3.92%)	247 (7.59%)	0.16	372 (6.45%)	226 (7.23%)	0.03
Hyperlipidaemia	573 (4.40%)	229 (7.04%)	0.11	396 (6.87%)	216 (6.91%)	0.00
Coronary artery disease	367 (2.82%)	166 (5.10%)	0.12	266 (4.61%)	149 (4.77%)	0.01
Osteoporosis	199 (1.53%)	79 (2.43%)	0.07	135 (2.34%)	71 (2.27%)	0.01
Stroke	259 (1.99%)	128 (3.93%)	0.12	183 (3.17%)	114 (3.65%)	0.03
Asthma	421 (3.23%)	179 (5.50%)	0.11	295 (5.12%)	169 (5.41%)	0.01
COPD	554 (4.25%)	257 (7.90%)	0.15	444 (7.70%)	236 (7.55%)	0.01
Chronic kidney disease	203 (1.56%)	155 (4.76%)	0.18	177 (3.07%)	127 (4.06%)	0.05
Chronic liver diseases	554 (4.25%)	408 (12.53%)	0.30	506 (8.78%)	351 (11.23%)	0.08
Hyperthyroidism	116 (0.89%)	85 (2.61%)	0.13	103 (1.79%)	72 (2.30%)	0.04
Thyroiditis	6 (0.05%)	7 (0.22%)	0.05	4 (0.07%)	4 (0.13%)	0.02
Pancreatitis	20 (0.15%)	18 (0.55%)	0.07	17 (0.29%)	13 (0.42%)	0.02
Affective psychosis	733 (5.63%)	353 (10.84%)	0.19	531 (9.21%)	321 (10.27%)	0.04
Ankylosing spondylitis	13 (0.10%)	15 (0.46%)	0.07	12 (0.21%)	12 (0.38%)	0.03
Inflammatory bowel disease	121 (0.93%)	51 (1.57%)	0.06	91 (1.58%)	46 (1.47%)	0.01
HIV	2 (0.02%)	2 (0.06%)	0.02	0 (0.00%)	0 (0.00%)	0.00
Antiphospholipid antibody syndrome	2 (0.02%)	53 (1.63%)	0.18	2 (0.03%)	1 (0.03%)	0.00
Transfusion-dependent thalassemia		356 (10.94%)	–		306 (9.80%)	

### Comparison of incidence and aHR of gout arthritis between thalassemia and controls

**Figure 2:**
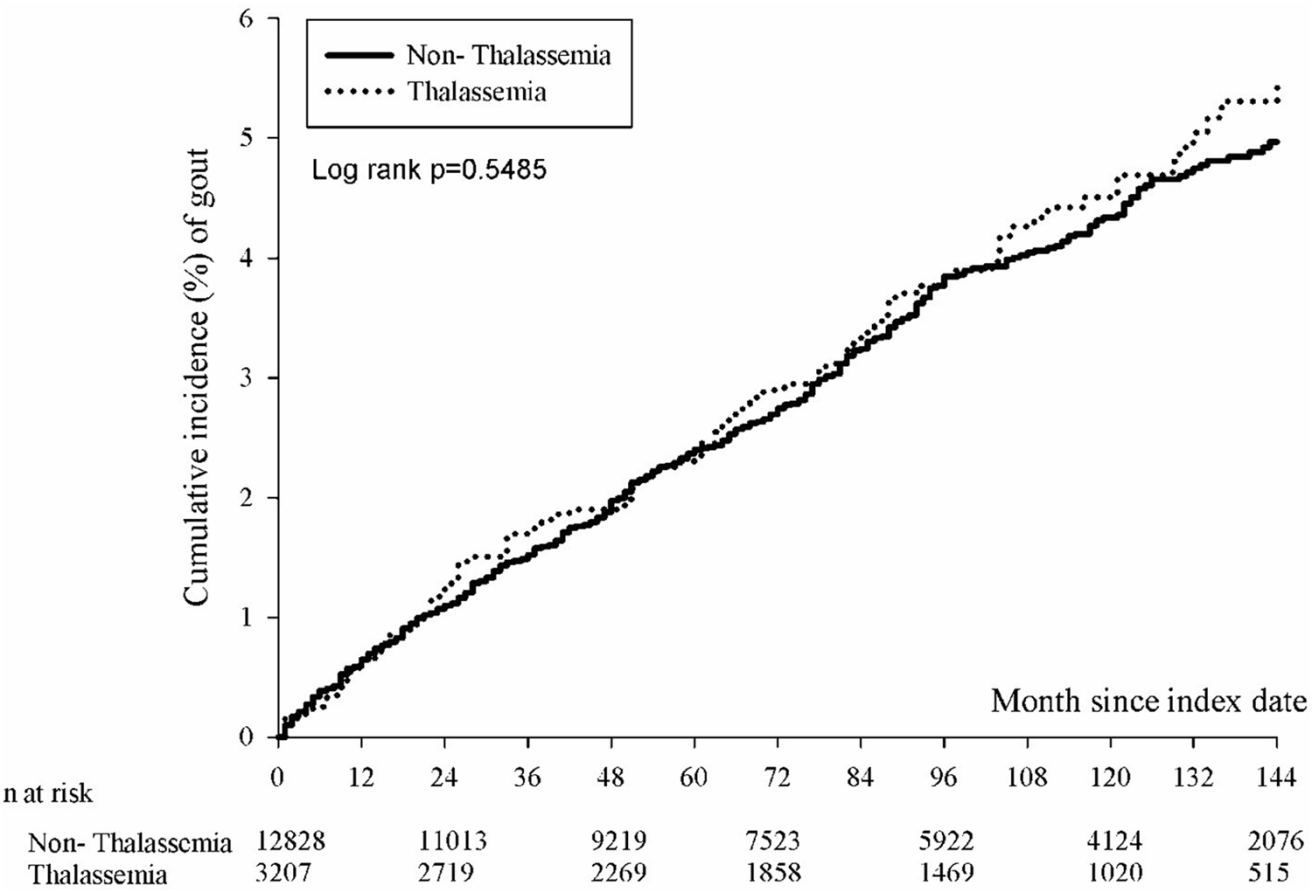
Cumulative Incidence of gout arthritis.

Table 2 showed the incidence and the hazard ratio of gout arthritis between thalassemia and controls. In the age and sex matched cohorts, the incidence of gout arthritis in the non-thalassemia group and the thalassemia group was 0.54 (95% CI 0.50 to 0.59) and 0.61 (95% CI 0.51 to 0.72) per 100 patients-year, respectively. After PSM, the incidence of gout arthritis in the non-thalassemia group and the thalassemia group was 0.48(95% CI 0.42 to 0.56) and 0.60(95% CI 0.51 to 0.72) per 100 patients-year, respectively. Before PSM, the crude hazard ratio of gout arthritis in the thalassemia group was 1.07 (95% CI 0.86 to 1.32) compared with the non-thalassemia group. After adjustment of demographic variables like sex, age, urbanization, and insured type, the adjusted hazard ratio (aHR) of gout arthritis in the thalassemia group was 1.09 (95% CI 0.88 to 1.34) compared with the controls. After adjustment of demographic variables and comorbidities, the aHR was 1.00 (95% CI 0.80 to 1.25). After PSM, the crude HR of gout arthritis in the thalassemia group was 1.11 (95% CI 0.87 to 1.42) compared with the control group. After adjustment of demographic data, the aHR of gout arthritis in the thalassemia group was 1.07 (95% CI 0.84 to 1.37) compared with the non-thalassemia group. After adjustment of demographic variables and comorbidities, the aHR was 1.09 (95% CI 0.85 to 1.39). The Kaplan–Meier curve showed that the cumulative incidence of gout arthritis was not a significant difference in the thalassemia group than in the comparison group (p = 0.5485) (Fig. 2).

**Table 2:** Incidence of gout in study groups†.

	Full cohort		PSM	
	Non-thalassemia n = 13,020	Thalassemia n = 3255	Non-thalassemia n = 5850	Thalassemia n = 3164
Median follow up time	88	87	89	87
Follow up person months	1,105,823	273,468	495,798	262,492
Incident event	500	138	200	132
Incidence rate*(95% CI)	0.54(0.50–0.59)	0.61(0.51–0.72)	0.48(0.42–0.56)	0.60(0.51–0.72)
Crude HR (95% CI)	Reference	1.07(0.86–1.32)	Reference	1.11(0.87–1.42)
aHR1 (95% CI )	Reference	1.09(0.88–1.34)	Reference	1.07(0.84–1.37)
aHR2 (95% CI)	Reference	1.00(0.80–1.25)	Reference	1.09(0.85–1.39)

*Incidence rate, per 100 patients-year.

^†^aHR1, adjusted hazard ratio, the co-variates including demographic variables (like sex, age, urbanization, and insured type).

aHR2, adjusted hazard ratio, the co-variates including demographic variables (such as sex, age, and insured type), and comorbidities.

### Univariable and multivariable Cox proportional hazard regression used to estimate the aHR of gout arthritis

Table 3 indicated the results of factors associated with the risk of gout arthritis in patients with and without thalassemia. The HR of gout arthritis for thalassemia exposure were 1.07 (95% CI 0.86 to 1.32) and 0.99 (95%: CI 0.78 to 1.24), through the univariable model and multivariable Cox regressions model. Both models showed that there was no significant risk of gout arthritis in thalassemia patients. In the multivariable model, males exhibited a higher risk of gout arthritis than did females (aHR 2.41, 95% CI 2.01 to 2.88). the older patients showed a 1.52-fold higher risk of gout arthritis compared with the young patients (aHR 1.52, 95% CI 1.13 to 2.04). The patients who had comorbidities with hypertension (aHR 1.75, 95%CI: 1.34 to 2.27), hyperlipidemia (aHR 1.45, 95% CI 1.06 to 1.99), and osteoporosis (aHR 1.77, 95% CI 1.11 to 2.81) exhibited a higher risk of gout arthritis.

**Table 3:** Estimation the hazard ratio of gout arthritis by using Cox proportional hazard regression before PSM.

	Univariate modeling		Multiple modeling	
	HR	95% CI	aHR	95% CI
Thalassemia (ref: non-Thalassemia)	1.07	0.86–1.32	0.99	0.80–1.24
Age at index date				
< 30	**0.46**	**0.37–0.59**	**0.39**	**0.30–0.49**
30–45	Reference		Reference	
45–60	**1.69**	**1.33–2.15**	**1.37**	**1.06–1.75**
≥ 60	**2.86**	**2.24–3.64**	**1.52**	**1.13–2.04**
Sex				
Female	Reference		Reference	
Male	**2.08**	**1.75–2.47**	**2.41**	**2.01–2.88**
Urbanization				
Urban	Reference		Reference	
Sub-urban	1.09	0.90–1.31	1.03	0.84–1.25
Rural	1.08	0.80–1.46	0.91	0.65–1.28
Insured unit				
Public insurance	Reference		Reference	
Labour insurance	1.19	0.83–1.72	1.29	0.90–1.86
Agricultural insurance	1.68	1.12–2.52	1.49	0.97–2.28
Low-income household	0.84	0.20–3.50	0.89	0.21–3.74
Company insurance	1.28	0.85–1.95	1.18	0.78–1.80
Other	0.62	0.27–1.39	0.74	0.33–1.69
Length of hospital stay				
0	Reference		Reference	
1–6	1.28	0.93–1.77	1.23	0.88–1.71
≥ 7	1.95	1.32–2.87	1.08	0.72–1.65
Co-morbidities				
Rheumatoid arthritis	1.27	0.41–3.96	0.74	0.23–2.38
Sjogren’s syndrome	3.06	1.14–8.18	2.17	0.79–5.96
Systemic sclerosis	–	–	–	–
Vasculitis	1.51	0.21–10.69	1.41	0.19–10.30
Hypertension	**3.99**	**3.24–4.91**	**1.75**	**1.34–2.27**
Diabetes mellitus	3.08	2.31–4.12	1.05	0.74–1.48
Hyperlipidaemia	**3.48**	**2.66–4.54**	**1.45**	**1.06–1.99**
Coronary artery disease	2.83	2.01–3.99	0.87	0.59–1.26
Osteoporosis	**2.99**	**1.91–4.67**	**1.77**	**1.11–2.81**
Stroke	3.35	2.24–5.00	1.09	0.70–1.69
Asthma	1.31	0.88–1.94	1.07	0.71–1.60
COPD	1.91	1.40–2.60	1.03	0.74–1.44
Chronic kidney disease	3.02	2.03–4.48	1.31	0.85–2.01
Chronic liver diseases	1.58	1.16–2.16	0.90	0.65–1.23
Hyperthyroidism	0.51	0.16–1.59	0.50	0.16–1.55
Thyroiditis	–	–	–	–
Pancreatitis	–	–	–	–
Affective psychosis	1.90	1.44–2.50	1.25	0.93–1.67
Ankylosing spondylitis	–	–	–	–
inflammatory bowel disease	1.44	0.72–2.90	1.39	0.69–2.80
HIV	–	–	–	–
Antiphospholipid antibody syndrome	1.78	0.57–5.54	2.09	0.66–6.61

### Sensitivity analysis under different definition of thalassemia and gout arthritis

The details of demographic information between individuals without thalassemia diagnosis, patients with non-transfusion-dependent thalassemia, and patients with transfusion-dependent thalassemia in Supplementary Table S1. In this study, the average $\pm$ SD of age at index date was 31.42 $\pm$ 17.57, 52.73 $\pm$ 22.06 and the female-to-male ratio was 1.68 and 1.27 in patients with non-transfusion-dependent thalassemia, and patients with transfusion-dependent thalassemia, respectively.

Supplementary Table S2 showed the incidence and hazard ratio of gout arthritis for non-transfusion thalassemia patients and transfusion-dependent thalassemia patients compared with non-thalassemia individuals.For primary outcome, that defined as diagnosis of gout arthritis (ICD-9-CM code 274.0) with more than 2 outpatient visits or at least one hospitalization, the crude hazard ratio was 0.94 (95% CI 0.76 to 1.17) and 2.13 (95% CI 1.37 to 3.29) in patients with non-transfusion thalassemia and patients with transfusion-dependent thalassemia, respectively. The hazard ratio adjusted with demographic variables (such as sex, age, and insured type) and comorbidities was 0.93 (95% CI 0.74 to 1.15) and 0.99 (95% CI 0.62 to 1.59) in patients with non-transfusion thalassemia and patients with transfusion-dependent thalassemia, respectively. For secondary outcome, that defined as diagnosis of gout arthritis and receiving antigout drugs, the crude hazard ratio was 0.92 (95% CI 0.72 to 1.18) and 2.40 (95% CI 1.50 to 3.85) in patients with non-transfusion thalassemia and patients with transfusion-dependent thalassemia, respectively. The hazard ratio adjusted with demographic variables (like sex, age, urbanization, and insured type) was 0.92 (95% CI 0.71 to 1.19) and 1.05 (95% CI 0.63 to 1.76) in patients with non-transfusion thalassemia and patients with transfusion-dependent thalassemia, respectively.

## Discussion

To our knowledge, this study was the first retrospective cohort study to evaluate the risk of gout arthritis, which was based on a comprehensive national databank. Our results demonstrated that patients with thalassemia did not have an increased risk of gout arthritis compared with the control population. Furthermore, we also observed that the male had a higher risk of gout arthritis compared with the female. The older patients exhibited a higher risk of gout arthritis than younger patients. The patients with thalassemia who had comorbidities with hypertension, hyperlipidemia, and osteoporosis showed a significant risk of gout arthritis.

There are few reports on the incidence of gouty arthritis in patients with thalassemia^15^. Recently, a cross-sectional study conducted in Thailand found that the prevalence of hyperuricemia in the thalassemia cohort was about 40%, but gouty arthritis accounts for only 6% of the study population^16^. The researchers proposed that the relatively high red blood cell turnover rate of thalassemia may be related to higher endogenous uric acid or serum uric acid (SUA)^2^. Similarly, this population-based retrospective cohort study discovered that the prevalence of gout arthritis in the thalassemia cohort was 5.05%, with aHR 1.00.

Several studies roughly reported the association between gout and thalassemia^7,11^. Paik et al.^7^ showed two cases of black patients with thalassemia and gouty arthritis. The high uric acid concentration, excessive UA excretion, and temporal connection in one of the patients indicated that gout was a secondary disease of hemoglobinopathy. Kumar et al.^11^ described a Greek subject with thalassemia related to end-stage kidney disease and gout. They confirmed the first case of crystal-proven gout in thalassemia patients and concluded that the long-term viability and kidney failure had inevitably invited the development of gout in this patient. As a result, It was vital to consider gout in the various diagnosis of patients with thalassemia and chronic arthritis. The red blood cell turnover rate of patients with thalassemia increased, and the final development of hyperuricemia was a predictable result^17^. The prevalence of inflammatory rheumatic diseases in patients with thalassemia disease was rather high. Positive direct Coombs test and antinuclear antibody were common in transfusion-dependent patients^18^. Hyperuricemia can lead to gout if it is poorly controlled over time, but clinical gout was rare in thalassemia and our research supported this conclusion. Unfortunately, the mechanism behind this association was still unclear. There is an urgent need for more research to clarify the possible biological mechanisms of them.

Prior research reported that hypertension, hyperlipidemia, and osteoporosis were more prevalent in gout arthritis patients. A prospective epidemiological study conducted in the United States showed that hypertension was a predictor of gout arthritis risk^19^. A systematic review and meta-analysis also revealed hypertension had a 1.64 times increased risk of gout compared to those in the normal population^20^. Our result following the above research results. A cohort study based on the Taiwanese population indicated that the risk rate of gout was significantly higher in men than in women, with OR 1.95, and hyperlipidemia was closely associated with the risk of gout (OR = 4.03)^21^. It is speculated that the abnormal regulation of carbohydrate in gout patients leads to the rise of blood lipid^22^. Likewise, our study supported that males were more likely to suffer from gout arthritis (aHR 2.41), and hyperlipidemia correlated with a greater risk of gout (aHR 1.45). A nationwide population-based retrospective cohort study showed that osteoporosis syndrome was associated with a 1.2-fold increase in gout arthritis outcomes and aHR was 1.20^23^. The cohort in Denmark and Taiwan also showed that the incidence of osteoporosis in the gout arthritis group increased by 1.25 times and 1.17 times, with aHR of 1.25 and 1.17^24,25^. Our result similarly concluded that osteoporosis (aHR 1.77) increased the risk of gout arthritis. The most likely explanation was that the accumulation of sodium urate crystals in the joint activated the NLRP3 inflammasome, and elevated levels of interleukin-1 (IL-1), IL-6, and tumor necrosis factor (TNF-alpha) may promote bone absorption^25^.

This study had the virtue of large sample data, population-based cohort, sufficient information on long-term cumulative incidence and comorbidities. However, there were several limitations to consider. First, the patients diagnosed with thalassemia and gout arthritis, were defined by ICD-9-CM code 282.4x, and 274.0 from 2000 to 2013 from LHID 2000. The diagnostic code of thalassemia were not validated in Taiwan NHIRD, and we cannot determine hemoglobin typing by using ICD-9-CM records. There is no laboratory result to identify the hemoglobin level and the hemoglobin typing by using medical claim datasets. However, the characteristic of thalassemia patients in Taiwan NHIRD was similar with the Korea study, that reported the prevalence of thalassemia was higher in female and elder population (reference: J. Clin. Med. 2022, 11, 2289). In the disease severity stratified analysis, the adjusted hazard ratio was 0.94 and 1.05 in patients with non-transfusion thalassemia and patients with transfusion-dependent thalassemia, respectively. We defined the alternative, secondary outcome for diagnosis of gout arthritis as the patient had ICD-9-CM code 274.0 and receiving antigout drugs, involved colchicine, cinchophen, allopurinol, febuxostat, and benzbromarone, the adjusted hazard ratio was 0.92 and 1.05 in patients with non-transfusion thalassemia and patients with transfusion-dependent thalassemia, respectively. Second, the LHID 2000 database did not provide more details on personal data such as smoking, drinking, biochemical indicators (hemolysis and serum uric acid level), and anti-hyperuricemic therapy, which restricted further research. Furthermore, patients from other ethnicities or countries were not included in this cohort to analyze this association.

## Conclusion

This study indicated that there was no significant increase in the risk of gout arthritis in subjects with thalassemia. Future detailed studies need to clarify the biological mechanisms behind this connection.

## Supplementary Information

Supplementary Table S1.

Supplementary Table S2.

## Data Availability

The datasets used and analysed during the current study available from the corresponding author on reasonable request.
