# Supplementary material for: Risk of gout attack not increased in patients with thalassemia: a population-based cohort study
Source: Sci Rep. 2023 Feb 16;13:2756. doi: 10.1038/s41598-023-29709-3 (PMC9935512; doi:10.1038/s41598-023-29709-3)
Supplement: Supplementary file 1 — Supplementary Table S1. [file 41598_2023_29709_MOESM1_ESM.docx]

Table S1 Characteristics among age and sex matched non-thalassemia group and thalassemia group stratified by transfusion-dependent

|  | Age and sex matched non-thalassemia group and thalassemia group | | |
| --- | --- | --- | --- |
|  | Non- thalassemia n= 13020 | Non-transfusion thalassemia n= 2899 | Transfusion-dependent thalassemia  n= 356 |
| **Age at index date** |  |  |  |
| <30 | 5842 (44.87%) | 1408 (48.57%) | 50 (14.04%) |
| 30-45 | 3483 (26.75%) | 798 (27.53%) | 78 (21.91%) |
| 45-60 | 2141 (16.44%) | 456 (15.73%) | 80 (22.47%) |
| ≥60 | 1554 (11.94%) | 237 (8.18%) | 148 (41.57%) |
| **Sex** |  |  |  |
| Female | 8076 (62.03%) | 1820 (62.78%) | 199 (55.90%) |
| Male | 4944 (37.97%) | 1079 (37.22%) | 157 (44.10%) |
| **Urbanization** |  |  |  |
| Urban | 8042 (61.77%) | 1750 (60.37%) | 187 (52.53%) |
| Sub-urban | 3781 (29.04%) | 895 (30.87%) | 123 (34.55%) |
| Rural | 1197 (9.19%) | 254 (8.76%) | 46 (12.92%) |
| **Insured unit** |  |  |  |
| Public insurance | 926 (7.11%) | 240 (8.28%) | 24 (6.74%) |
| Labour insurance | 8121 (62.37%) | 1845 (63.64%) | 180 (50.56%) |
| agricultural insurance | 1690 (12.98%) | 343 (11.83%) | 86 (24.16%) |
| Low-income household | 69 (0.53%) | 17 (0.59%) | 6 (1.69%) |
| Company insurance | 1866 (14.33%) | 385 (13.28%) | 54 (15.17%) |
| Other | 348 (2.67%) | 69 (2.38%) | 6 (1.69%) |
| **Length of hospital stay** |  |  |  |
| 0 | 12080 (92.78%) | 2499 (86.20%) | 195 (54.78%) |
| 1-6 | 629 (4.83%) | 258 (8.90%) | 52 (14.61%) |
| ≥7 | 311 (2.39%) | 142 (4.90%) | 109 (30.62%) |
| **Co-morbidities** |  |  |  |
| Rheumatoid arthritis | 65 (0.50%) | 32 (1.10%) | 8 (2.25%) |
| Sjogren’s syndrome | 44 (0.34%) | 18 (0.62%) | 0 (0.00%) |
| Systemic sclerosis | 0 (0.00%) | 1 (0.03%) | 0 (0.00%) |
| Vasculitis | 10 (0.08%) | 8 (0.28%) | 1 (0.28%) |
| Hypertension | 1090 (8.37%) | 268 (9.24%) | 132 (37.08%) |
| Diabetes mellitus | 511 (3.92%) | 160 (5.52%) | 87 (24.44%) |
| Hyperlipidaemia | 573 (4.40%) | 180 (6.21%) | 49 (13.76%) |
| Coronary artery disease | 367 (2.82%) | 113 (3.90%) | 53 (14.89%) |
| Osteoporosis | 199 (1.53%) | 55 (1.90%) | 24 (6.74%) |
| Stroke | 259 (1.99%) | 73 (2.52%) | 55 (15.45%) |
| Asthma | 421 (3.23%) | 151 (5.21%) | 28 (7.87%) |
| COPD | 554 (4.25%) | 189 (6.52%) | 68 (19.10%) |
| Chronic kidney disease | 203 (1.56%) | 83 (2.86%) | 72 (20.22%) |
| Chronic liver diseases | 554 (4.25%) | 321 (11.07%) | 87 (24.44%) |
| Hyperthyroidism | 116 (0.89%) | 80 (2.76%) | 5 (1.40%) |
| Thyroiditis | 6 (0.05%) | 7 (0.24%) | 0 (0.00%) |
| Pancreatitis | 20 (0.15%) | 14 (0.48%) | 4 (1.12%) |
| Affective psychosis | 733 (5.63%) | 290 (10.00%) | 63 (17.70%) |
| Ankylosing spondylitis | 13 (0.10%) | 13 (0.45%) | 2 (0.56%) |
| inflammatory bowel disease | 121 (0.93%) | 46 (1.59%) | 5 (1.40%) |
| HIV | 2 (0.02%) | 2 (0.07%) | 0 (0.00%) |
| Antiphospholipid antibody syndrome | 2 (0.02%) | 47 (1.62%) | 6 (1.69%) |
