# Supplementary material for: Risk of gout attack not increased in patients with thalassemia: a population-based cohort study
Source: Sci Rep. 2023 Feb 16;13:2756. doi: 10.1038/s41598-023-29709-3 (PMC9935512; doi:10.1038/s41598-023-29709-3)
Supplement: Supplementary file 2 — Supplementary Table S2. [file 41598_2023_29709_MOESM2_ESM.docx]

Table S2 Incidence and hazard ratio of gout arthritis in study groups†

|  | Age and sex matched non-thalassemia group and thalassemia group | | |
| --- | --- | --- | --- |
|  | Non- thalassemia n= 13020 | Non-transfusion thalassemia n= 2899 | Transfusion-dependent thalassemia  n= 356 |
| Primary outcome: diagnosis of gout arthritis (ICD-9-CM code 274.0) with more than 2 outpatient visits or at least one hospitalization |  |  |  |
| Median follow up time | 88 | 88 | 48 |
| Follow up person months | 1105823 | 251260 | 22208 |
| Incident event | 500 | 115 | 23 |
| Incidence rate*(95% C.I.) | 0.54(0.50-0.59) | 0.54(0.46-6.57) | 1.24(0.81-1.84) |
| Crude HR (95% C.I.) | Reference | 0.94(0.76-1.17) | 2.13(1.37-3.29) |
| aHR1 (95% C.I.) | Reference | 1.06(0.86-1.32) | 1.23(0.79-1.90) |
| aHR2 (95% C.I.) | Reference | 0.93(0.74-1.15) | 0.99(0.62-1.59) |
| Secondary outcome: diagnosis of gout arthritis (ICD-9-CM code 274.0) and receiving antigout drugs |  |  |  |
| Median follow up time | 88 | 88 | 48 |
| Follow up person months | 1120394 | 252525 | 22290 |
| Incident event | 309 | 64 | 15 |
| Incidence rate*(95% C.I.) | 0.33(0.30-0.37) | 0.30(0.24-0.39) | 0.81(0.47-1.30) |
| Crude HR (95% C.I.) | Reference | 0.92(0.72-1.18) | 2.40(1.50-3.85) |
| aHR1 (95% C.I.) | Reference | 1.06(0.83-1.36) | 1.31(0.82-2.12) |
| aHR2 (95% C.I.) | Reference | 0.92(0.71-1.19) | 1.05(0.63-1.76) |

* Incidence rate, per 100 patients-year

†

aHR1, adjusted hazard ratio, the co-variates including demographic variables (like sex, age, urbanization, and insured type).

aHR2, adjusted hazard ratio, the co-variates including demographic variables (such as sex, age, and insured type), and comorbidities.
